# Structural and Exchange Components in Processes of Neighbourhood Change: A Social Mobility Approach

**DOI:** 10.1007/s12061-017-9249-z

**Published:** 2018-01-19

**Authors:** Tal Modai-Snir, Maarten van Ham

**Affiliations:** 1 OTB - Research for the Built Environment, Faculty of Architecture and the Built Environment Delft University of Technology, PO Box 5043, 2600GA Delft, The Netherlands; 2 School of Geography & Sustainable Development, Irvine Building, University of St Andrews, North Street, St Andrews, Fife, Scotland KY16 9AL UK

**Keywords:** Urban change, Neighbourhood change, Structural processes, Relative change, Absolute change, Inequality

## Abstract

Neighbourhood socioeconomic change is a complex phenomenon which is driven by multiple processes. Most research has focused on the role of urban-level processes, which lead to an exchange of relative positions among neighbourhoods of a single metropolitan area. Consequently, the effects of structural processes on neighbourhood socioeconomic change, such as overall income growth or decline, and increasing inequality, have been neglected. This is reflected in the standard methodological practices; the common measures of neighbourhood change exclude the effect of overall growth or decline and confound the effects of urban processes with the effect of increase in inequality. This paper proposes a method that was originally developed for understanding income mobility of individuals, to decompose total neighbourhood socioeconomic change measured in absolute terms into its contributing components. The approach enables to take account of all processes that generate neighbourhood socioeconomic change, while distinguishing between them. The method is demonstrated in an empirical analysis of neighbourhood socioeconomic change across 22 metropolitan areas in the US. The findings indicate that structural processes can be most substantial in generating change. Neighbourhood socioeconomic change in ‘superstar cities’ is mostly generated by the growth in overall incomes, with a relatively low contribution of increasing inequality. Conversely, in declining cities it is mostly driven by overall decline and increasing inequality. An additional finding relates to the interaction between urban processes and increasing inequality. These processes work in opposite directions such that any increase in positions of low-income neighbourhoods can be totally offset by an income decrease due to increasing inequality.

## Introduction

The socioeconomic status of urban neighbourhoods can change over time due to multiple processes. Some of these processes operate at the urban level, which cause neighbourhoods to change relative to one another within the urban area. The movement of neighbourhoods up and down the urban socioeconomic hierarchy is often related to their life cycle and stage of development; many neighbourhoods decline as they age, and at some point might be subject to renewal processes which lead to an upward trajectory (Hoover and Vernon 1959). Much of this cyclical movement is associated with the deterioration of the housing stock and its consequent filtering down to lower-income households (Muth 1973; Sweeney 1974a, 1974b). Social dynamics also drive these changes, for example, households’ preferences for living among similar households generate sorting among neighbourhoods, as demonstrated by Schelling's (1971) segregation model. Institutional actions at the urban level can change neighbourhoods’ socioeconomic positions as well. Mainly, such actions include interventions in the housing stock (e.g. Andersson and Musterd 2005), public investments in urban amenities (Van Criekingen and Decroly 2003) or environmental improvements (Meen et al. 2012).

Beyond the urban level, neighbourhood socioeconomic conditions are also affected by structural processes that involve regional, national and global levels. These processes can generate changes in the distribution of socioeconomic characteristics of the population in an urban area, which can translate into neighbourhood change. Overall socioeconomic levels can increase or decrease at the regional or national level, and consequently they can increase or decrease at the neighbourhood level (Galster et al. 2003; Zwiers et al. 2016a). Also changing levels of inequality in society can affect neighbourhoods (Andersson and Hedman 2016).

These various processes can be summarised into three distinct effects on changes in neighbourhood socioeconomic conditions (Collver and Semyonov 1979). Urban-level processes generate an ‘exchange’ effect which implies that over time, neighbourhoods switch relative positions in their urban hierarchy. It is a zero-sum process that simply circulates advantage and disadvantage among urban neighbourhoods, within a given distribution of neighbourhood average incomes. Two different ‘structural’ effects, on the other hand, change the distribution of neighbourhood average incomes in the urban area: The ‘growth/decline’ effect which increases or decreases incomes among all neighbourhoods in the urban area, and the ‘inequality’ effect which increases the disparities among them. Globalization and re-urbanization processes in the last decades generated increasing inequality within and between urban areas. Wealth has increasingly concentrated in fewer ‘superstar’ cities while others have been left behind (Florida 2017; Gyourko et al. 2006); this points to the importance of the ‘growth/decline’ effect on neighbourhoods. Urban areas have become more polarized, with more rich and poor neighbourhoods and less middle-income neighbourhoods (Booza et al. 2006; Florida 2017; Hulchanski 2010; Tammaru et al. 2015), which emphasizes the role of the ‘inequality’ effect in generating neighbourhood upward or downward change.

The way neighbourhood change has been measured in most research on neighbourhood socioeconomic change does not enable to distinguish between these different components of change. The commonly used measures (Choldin and Hanson 1982; Delmelle 2015; Fogarty 1977; Gould Ellen and O’Regan 2008; Landis 2016; Logan and Schneider 1981; Owens 2012; Rosenthal 2008; Rosenthal and Ross 2015) leave out the overall change in incomes throughout the urban areas. They do, nevertheless, capture the effect of increasing inequality among neighbourhoods, but then confound it with the ‘exchange’ effect related to urban processes. Ultimately, most existing research mainly focused on changes that neighbourhoods experience relative to others in the same urban area (what we call the ‘exchange’ effect). Neither the effect of overall growth and decline, nor the effect of changing inequality levels gained enough attention, despite their increasing role in understanding socio-spatial divisions in recent decades. The processes of growth and decline and inequality have become so powerful, that we believe that their effect on neighbourhood socioeconomic conditions can overshadow that of urban processes, which have traditionally gained more attention in the literature. Currently, however, we know very little about the relative roles of the three processes in understanding the changing urban hierarchy.

The objective of this paper is to develop an approach to measure neighbourhood socioeconomic change that can be used to investigate and distinguish all the contributing processes. Such an approach will enable us to look at neighbourhood change from a wider perspective, including not only the urban context but also higher-level contexts which have become, we suspect, at least as important in determining neighbourhood fortunes. The paper proposes a method to understand neighbourhood change which comes from studies of income mobility.

Social and income mobility research have long been making the distinction between exchange and structural components with regard to mobility measurement. Two previous studies have already applied a similar approach in the neighbourhood and urban context (Collver and Semyonov 1979; Congdon and Shepherd 1988), partially based on the work of McClendon (1977). This paper, however, challenges the conceptual underpinnings of the components derived by these authors. This paper aims to advance the approach by considering more recent methodological contributions in income mobility research. Specifically, this paper uses a decomposition method presented by Van Kerm (2004). The paper ends with an empirical analysis of neighbourhood socioeconomic change by using real data from US metropolitan areas.

## Theoretical Background

The changing mosaic of neighbourhoods and their socioeconomic status hierarchy has fascinated researchers for long, and has resulted in a large literature which reflects the complexity of processes of change. One strand of the literature focuses on explaining the sources of change through an individual-level approach. This approach examines how residential mobility, social mobility and demographic changes alter the socioeconomic compositions of neighbourhoods (Bailey et al. 2017; C. Hochstenbach and Musterd 2017; Cody Hochstenbach and van Gent 2015; Teernstra 2014). Individual-level studies focus, therefore, on how neighbourhood change is realized through the aggregate changes in individual social and spatial positions. A different approach can be characterized as a system-level one, which focuses on neighbourhoods as parts of an urban socioeconomic hierarchy. This approach focuses on the underlying factors that generate change through their effect on individuals. For example, how the evolution of cities, economic and societal trends and institutional actions affect neighbourhoods. This paper is located within the system-level perspective on neighbourhood and urban change. The following section on theoretical background reviews diverse theories that deal with the question of why neighbourhoods change from a systems perspective. We sort the various factors into those pertaining to urban dynamics (termed ‘exchange’ processes) and those which are related to structural processes (‘growth/decline’ and ‘inequality’) involving the regional, national and global levels.

### Neighbourhood Change and Urban-Level Processes

An influential class of urban models depicted neighbourhood socioeconomic change as a cyclic process. The early ‘invasion-succession’ model developed by Chicago School sociologists (Park 1952) suggested that low-income households take the place of higher-income households who gradually move outward to newer neighbourhoods at the urban fringe. Two other models complement this view; the Life cycle model (Hoover and Vernon 1959) suggests that neighbourhoods move chronologically through stages of development, characterized by gradual decline, until they reach a point that reinvestment is economically worthy and go through a process of renewal. The filtering model (Muth 1973; Sweeney 1974a, 1974b) emphasizes the role of the deterioration of the neighbourhood’s housing stock in generating neighbourhood decline. It drives away affluent households to newer neighbourhoods while the vacated housing filters down to lower-income households. Empirical studies asserted, in general, the life-cycle and filtering view (e.g. Brueckner and Rosenthal 2009; Choldin et al. 1980; Choldin and Hanson 1982; Rosenthal 2008; Rosenthal and Ross 2015), indicating a pattern of mean reversion; high-income neighbourhoods typically experience decline while low-income ones experience increase. In a study that analysed unique historical data of Philadelphia County, Rosenthal (2008) found that it took roughly 100 years for the city’s neighbourhoods to cycle back to their initial income level.

Other urban-development processes can also explain neighbourhood socioeconomic change, regardless of the life-cycle stage. Transportation innovations such as commuter networks, for example, have found to be one of the drivers of the historical flight of high- and middle- income households to the suburbs (Anas et al. 1998), with its long-lasting effects on city-centre decline in many metropolitan areas, especially in the US. The suburbanization of employment has also contributed to that decline (Wilson 1987). Later evidence associated public investment in rail transit systems with gentrification and socioeconomic increase of certain neighbourhoods (Kahn 2007). Other public investments can also generate gentrification and upgrading, for example, environmental improvements (Meen et al. 2012) and public investment in historical areas (Van Criekingen and Decroly 2003). Also urban conservation practices and policies can bring such impact (Lees 1994). Other urban policies aim at generating socioeconomic upgrades through physical changes to the housing stock; the outcomes of such restructuring policies are often the displacement of low-income households from deprived neighbourhoods (Andersson and Bråmå 2004; Andersson and Musterd 2005; Bolt and van Kempen 2010). Common criticism related to such policies is that problems associated with poverty do not disappear due to such interventions, but move to other places within the urban area (Andersson and Musterd 2005). This depiction, of ‘moving disadvantage’ can be generalized to all income strata, as well as to other driving mechanisms. As long as population characteristics do not change, urban-level processes simply move advantage and disadvantage and cause the exchange of positions among urban neighbourhoods.

The preference of people for living among people similar to themselves is central in generating neighbourhood change, as demonstrated in Schelling's (1971) seminal model. The model shows that even slight preferences for own-group presence can drive such change and lead to segregation. Social dynamics are self-reinforcing as the increasing presence of own-group households further attracts similar households; thus, they can either accelerate the pace of socioeconomic change or make status persistent (Rosenthal 2008; Rosenthal and Ross 2015). Housing market dynamics also play a role in reinforcing the process of change, as changes are quickly manifested in housing prices. The literature on gentrification, for example, describes how an initial inflow of high-income households can increase housing prices and trigger the displacement of existing low-income residents (e.g. Atkinson 2000; Marcuse 1986). Some local amenities, such as retail and public services, also take part in these dynamics; their location reflects the presence of certain socioeconomic strata in the neighbourhood, but at the same time they further attract other households of similar status (Glaeser and Gyourko 2005; Rosenthal and Ross 2015).

Although socioeconomic change is most common, there are neighbourhoods that persistently occupy a stable relative position in the urban neighbourhood hierarchy (Rosenthal 2008). Rosenthal’s study (2008), indicated that a third of all neighbourhoods remained in the same income quartile over a period of 50 years. Delmelle (2017) rather identified stability as the most frequent pathway among US metropolitan neighbourhoods, but this finding relies on a different definition of stability. Some urban features explain persistence in neighbourhood relative status. Landscape features (Lee and Lin 2013; Meen et al. 2012) and historical city centres (Brueckner et al. 1999), for example, represent fixed advantages that can be associated with persistent affluence. Negative features, such as environmental problems or inferior accessibility, can cause persistent deprivation.

### Neighbourhood Change and Structural Processes

Regardless of the repositioning of neighbourhoods within the urban hierarchy, various processes can drive changes in the absolute socioeconomic conditions of neighbourhoods. These processes, which are termed hereafter as structural, operate beyond the urban level and affect neighbourhood absolute conditions by changing the socioeconomic makeup of the metropolitan population. One of them, is the upward or downward change in overall socioeconomic conditions (termed hereafter the ‘growth/decline’ effect). neighbourhood changes can result from overall income growth or decline which follows from macro-economic and demographic processes throughout the country or in specific regions. In rust-belt metropolitan areas in the US, for instance, neighbourhood socioeconomic decline mirrored the decline of whole cities due to the shrinking of the industrial sector (Rosenthal and Ross 2015). Similarly, increasing poverty at the neighbourhood level was found to be most dependent on the increase in poverty in the surrounding county (Galster et al. 2003). Beyond the regional level, Zwiers et al. (2016b) illustrated how global processes, such as the 2008 crisis, may translate into decline among individual neighbourhoods. The ‘growth/decline’ effect means that the whole distribution of neighbourhood average incomes shifts upward or downwards.

Another type of structural process that can affect socioeconomic conditions of individual neighbourhoods is the change in the dispersion of the neighbourhood income distribution within an urban area. Such change is likely to result from changing economic inequality among individuals in the region or in society as a whole (hence termed the ‘inequality’ effect). Increasing inequality among individuals results in increasing disparities among neighbourhoods due to two different mechanisms (Andersson and Hedman 2016); first, when incomes of the rich and poor diverge, the average incomes of their respective places of residence follow the same path through an in situ process. Secondly, increasing income inequality generates intensified selective mobility because of the increased disparities between the rich and poor in the resources available to spend on housing. For example, in the US, increasing income segregation has been associated with increasing inequality among individuals (Reardon and Bischoff 2011; Watson 2009). Also, the decline in the proportion of middle-income neighbourhoods seems to correspond to a similar decline in the proportions of middle-income families in the overall population (Booza et al. 2006).

**Fig. 1:**
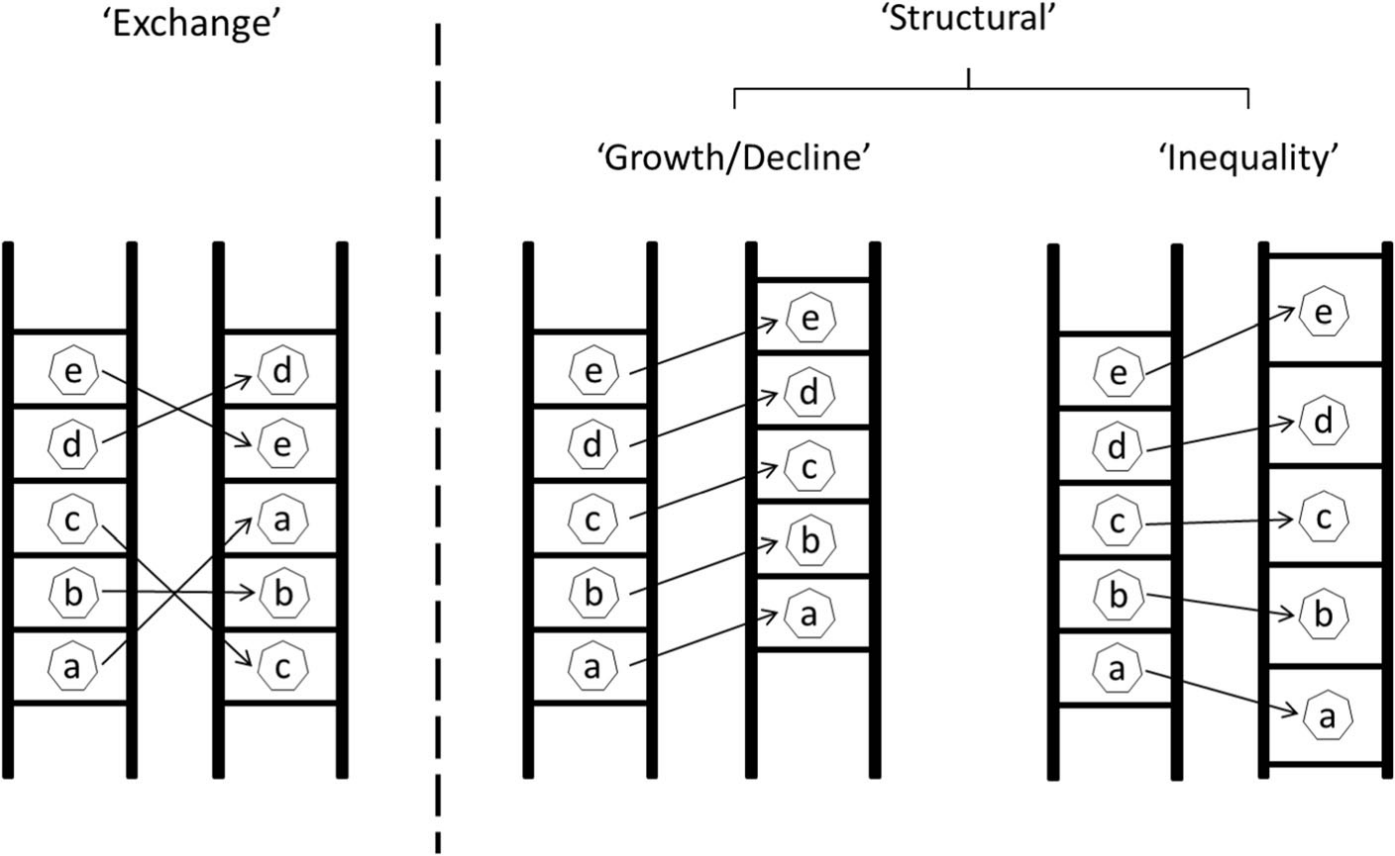
A conceptual distinction among processes of neighbourhood socioeconomic change. Source: authors

To summarize, neighbourhood socioeconomic change is a result of distinct processes operating at different levels: the urban level, which is associated with the ‘exchange’ effect, and higher (inter-regional, national or global) levels which are associated with two structural effects: The ‘growth/decline’ and ‘inequality’ effects. Figure 1 explains this distinction by illustrating the metropolitan socioeconomic hierarchy of neighbourhoods as a ladder. Each echelon signifies a certain socioeconomic position within the metropolitan hierarchy, occupied by a certain neighbourhood at each point in time. Each pair of ladders denotes a transition from one point in time to another, over which one can observe the changes occurring to the whole array of neighbourhoods and to each individual one. The left scheme illustrates the pattern of changes occurring among neighbourhoods due to the exchange of relative positions. The socioeconomic statuses incurred by each position on the ladders are identical across the two observations, and neighbourhoods just swap places among themselves. The middle scheme depicts the kind of change expected during a period of income growth. The socioeconomic level entailed by each position is higher at the second observation. During a period of overall decline socioeconomic levels among all positions would be lower. The right scheme visualizes the effect of changing inequality on neighbourhood change. In this example the distribution widens such that high-positioned neighbourhoods experience an increase of socioeconomic levels and low-positioned neighbourhoods experience a decrease. The opposite could happen if the distribution became more equal; the ladder scheme would depict positions which are closer to the average level, with smaller socioeconomic gaps among positions.

### Current Measures of Neighbourhood Change and their Limitations in Reflecting the Complexity of Processes

There are various ways to measure neighbourhood status change, and each captures a different combination of the ‘exchange’, ‘growth/decline’ and ‘inequality’ processes of change. Many studies measured neighbourhood change based on the status of neighbourhoods relative to their respective metropolitan area (Choldin and Hanson 1982; Delmelle 2015; Fogarty 1977; Gould Ellen and O’Regan 2008; Landis 2016; Logan and Schneider 1981; Owens 2012; Rosenthal 2008; Rosenthal and Ross 2015; Teernstra 2014). These measures eliminate the effect of metropolitan income growth or decline. So, if a neighbourhood is located in an economically declining or growing urban area, the absolute socioeconomic change implied by this process will not be captured when a relative measure is used. Relative measures understate, therefore, the upward or downward amount of change (Gould Ellen and O’Regan 2008; Jun 2013) and their use results in overlooking an important source of divergence in neighbourhoods’ conditions across metropolitan areas.

Neighbourhood socioeconomic change has also been measured based on status relative to other reference levels, for example, to the average of a cross-metropolitan sample of neighbourhoods (Jun 2013; Zwiers et al. 2016b). By using this reference level, measures account for processes that affect the disparities in growth or decline among the urban areas included in the sample. However, other structural processes that lead to overall growth or decline may still not be accounted for; for example, changing income disparities among metropolitan and rural areas or among sampled and non-sampled metropolitan areas, and a national growth/decline in incomes. Measuring neighbourhood change relative to the average of a national sample of neighbourhoods may account for all structural processes except a national increase or decline in incomes.

**Fig. 2:**
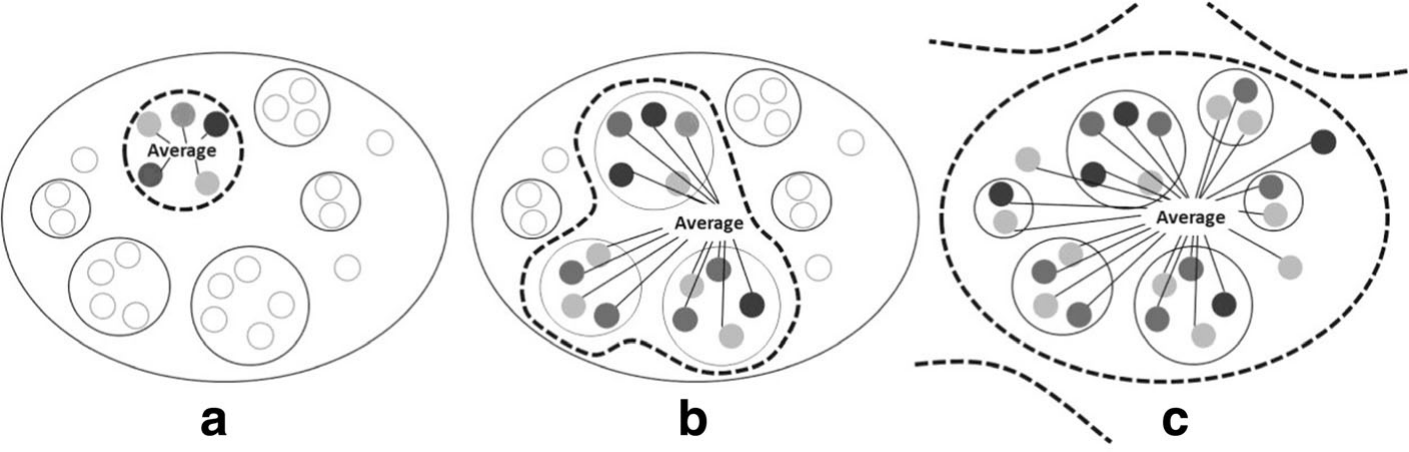
Using different spatial scales as reference levels in neighbourhood change measures. Source: authors

The higher the spatial scale used as a reference for neighbourhood status measurement, the more processes of change can be captured. Figure 2 illustrates that principle in three different cases (*a,b,c*). In each of the cases the outer boundary represents a whole region or a country, smaller circles represent metropolitan areas or cities and grey spots represent the smallest spatial units, referring to neighbourhoods. In case *a* neighbourhood change is measured based on status relative to the city- or metropolitan average; thus it only captures the effect of processes operating within the respective boundaries. Case *b* represents a situation where the reference level is the average of neighbourhoods across a sample which includes several cities or metropolitan areas. Consequently it captures processes that produce disparities among the sampled spatial units but overlooks those that may produce spatial disparities among sampled and non-sampled areas. Finally, case *c* shows that a reference level of a regional or country average captures all processes within that boundary; however, processes that operate beyond that level are still left out. Only a measure that is based on absolute income values would capture the overall amount of neighbourhood change associated with growth or decline processes. Using them, however, cannot indicate whether neighbourhoods changed relative to other metropolitan neighbourhoods or whether their change is related to the overall metropolitan, regional, or national increase or decline, and this is why relative measures have been used in the first place; they were assumed to isolate urban-level from higher-level structural processes (Logan and Schneider 1981).

However, the most common relative measures do not completely control for higher-level structural processes. Measures that are based on computing the ratio of neighbourhood average income to the average for all neighbourhoods in the respective metropolitan area (e.g. Fogarty 1977; Gould Ellen and O’Regan 2008; Logan and Schneider 1981; Rosenthal 2008; Rosenthal and Ross 2015), and to a lesser extent also those that are based on standardized scores (Delmelle 2015, 2017) do in fact capture the ‘inequality’ effect and therefore confound it with the ‘exchange’ effect. This can lead to the inconsistency of research designs with theoretical models, because the effect of changing inequality on neighbourhoods is incorporated in the total observed change which is attributed to urban-level processes. For example, increasing income inequality is expected to increase the absolute socioeconomic levels of the highest-positioned neighbourhoods and decrease those of the lowest status ones. This pattern can counteract the typical mean-reversion pattern associated with urban filtering, where high-income neighbourhoods move down and low-income ones move up. In that case, the amount of change attributed to urban-level processes can be understated.

It follows that most of the neighbourhood change literature neglected the overall effect of higher-level structural processes and also confounded different processes in their analyses. To fully account for all structural processes absolute measures should be used. But at the same time, the contributions of different processes of change have to be distinguished from each other to be able to compare neighbourhood change between, for example, different cities, and to be able to examine theoretical models that focus on specific sources of change. Two previous studies, suggested approaches that comply with this strategy. They decomposed total neighbourhood and urban change (measured in absolute terms) into contributing components (Collver and Semyonov 1979; Congdon and Shepherd 1988). Although we have some reservations about the conceptual implications underlying the derived components (which are discussed in the next section), the approach appears beneficial. This methodological direction has, nevertheless, not been further advanced.

This paper follows this abandoned route of neighbourhood change research; it proposes the application of an alternative decomposition procedure of total neighbourhood change to components reflecting ‘exchange’ and two different ‘structural’ effects: ‘growth/decline’ and ‘inequality’. The approach builds on methodological advancements in decomposing total mobility to its contributing ‘exchange’ and ‘structural’ components from the field of individual income mobility.

## A Social Mobility Approach to Decomposing Total Neighbourhood Change

The research field of social and income mobility of individuals also struggles with the decomposition of total mobility into structural- and exchange-mobility components. This paper proposes to use such a method and apply it to neighbourhood change research.

Social mobility deals with the changes in individuals’ social and economic positions through time. Sociologists have typically focused on transitions between parent’s and offspring’s’ socio-occupational positions. In this context, structural mobility has been referred to as the class mobility of individuals that is induced by the changing availability of occupational positions across class categories, due to technological development or other structural processes. Exchange mobility has been regarded as the movement of individuals among positions within a given distribution of positions among social classes (Markandya 1982). Welfare economists are focused on the evolution of economic well-being; with incomes at the centre of attention, the field is more specifically termed ‘income mobility’. Here, structural mobility refers to changes in individuals’ incomes which result from changes in the distribution of income, and exchange mobility is referred to as the change in individuals’ relative positions within a given distribution of incomes (Markandya 1982).

In the social and income mobility research there is a lack of consensus as to whether structural mobility matters. For example, welfare economists are divided by those who consider the change in individuals’ incomes resulting from overall growth as mobility, and those who do not. The latter, referred to as taking a relativist approach, would argue that substantial mobility only occurs if individuals experience change in relative positions across the income distribution [see Fields 2008 for a more detailed explanation]. It is agreed, however, that exchange and structural effects have to be distinguished from each other. Basically, this is done by quantifying the total amount of mobility and by decomposing it to reflect the contributions of the different effects. Yet, there are several distinctive conceptualizations of mobility (Fields 2008; Fields and Ok 1999a), and correspondingly, there are also different ways to measure it and to reflect its underlying components. Silber (1995), for example, presented a decomposition of total distributional change to a component generated by inequality change and a component reflecting the exchange of positions. The ‘total mobility’ decomposed in this case adheres to a relative concept of mobility and thus it excludes the ‘growth/decline’ component which we, in the context of this paper, do seek to account for. Another procedure, proposed by Ruiz-Castillo (2004), decomposes the mobility measure by Chakravarty et al. (1985) which is based on an ethical approach to mobility. The concept underlying this measure does not suit, in our opinion, the analysis of neighbourhood change. These two methods are therefore not applicable in the context presented in this paper.

Two papers introduced variants of another decomposition strategy and applied it in the context of neighbourhood and urban change (Collver and Semyonov 1979; Congdon and Shepherd 1988); both are partially based on previous work of McClendon (1977). They decompose the sum of squared differences between final and initial neighbourhood indicator values, and derive three similar components of change defined as 1) changes in the average over all neighbourhoods or areas 2) changes in the dispersion of the distribution of indicator values and 3) changes in the relative positions of neighbourhoods. These components can be regarded as the equivalents of the ‘growth/decline’, ‘inequality’ and ‘exchange’ effects respectively. The first component is expressed in both papers as the difference between final and initial overall means. This statistic is inconvenient in a comparative context, where a ratio statistic would be preferable. The second component is expressed by Collver & Semyonov as the difference in standard deviations of final and initial distributions. Congdon and Shepherd’s respective component is based on the beta coefficient computed from regressing final on initial indicator values and therefore it is also dependant on the relationship between final and initial standard deviations. Standard deviations are scale-variant and translation-invariant; scaling a variable changes the standard deviation proportionately, and adding a constant amount to a variable does not change the statistic. This is contrary to the axioms underlying the most commonly used inequality measures (for example the Gini index). Finally, the exchange component is expressed by Collver & Semyonov as the difference in standardized scores. The change in standardized scores is not void of structural influences.^1^ In case of significant change in the shape of the distribution standardized scores are affected and therefore do not ‘purely’ account for exchange processes. Our suggestion of applying an alternative decomposition rests therefore on the grounds of conceptual perception regarding the derived components.

## A Method for Decomposing Total Neighbourhood Change into Structural and Exchange Components

We propose the application of a decomposition presented by Van Kerm (2004). This procedure decomposes mobility measures to represent the relative contributions of ‘growth/decline’, ‘inequality’ (termed ‘dispersion’ by Van Kerm) and ‘exchange’ components of income mobility processes. It has the advantage of offering a general framework that can be applied to different mobility measures which could be chosen to conform to a particular research context. The method involves the analysis of two observations of a vector of neighbourhood average incomes (given in absolute terms), which represents a single, whole metropolitan system; the first observation is at time *t* and the second at time *t + 1.*The procedure is based on the construction of counterfactual income vectors, each representing the hypothetical effect of only one factor on the initial vector of incomes, while the effect of the other two factors is neutralized. Van Kerm (2004) specified three functions that can be used to derive the three counterfactual vectors. We explain the underlying rational, referring to the context of neighbourhood change [refer to Van Kerm 2004 for the technical overview and formulas which relate to the income mobility context].

1. The ‘exchange’ counterfactual vector illustrates how neighbourhood average incomes in the metropolitan area would look like if they were not affected by growth/decline or increasing inequality and only affected by the changes in relative positions. It is constructed by replicating the vector of neighbourhood average incomes observed at time *t*, but reordered according to the rank orders of neighbourhood average incomes at time *t + 1*.
2. The ‘structural’ counterfactual vector represents how neighbourhood average incomes in the metropolitan area would look like if neighbourhoods didn’t change relative positions and would only be affected by the growth/decline of incomes and changes in inequality. It is constructed by replicating the vector of neighbourhood average incomes observed at time *t + 1*, but reordered according to neighbourhood original rank orders that was observed at time *t*. While this counterfactual vector represents the combined impact of the two ‘structural’ components (‘growth/decline’ and ‘inequality’), the following two vectors represent the isolated effect of each.
3. The ‘growth/decline’ counterfactual vector is constructed by ‘inflating’ (or ‘deflating’) the vector of neighbourhood average incomes observed at time t by the ratio between the overall averages of *t + 1* and *t* neighbourhood average incomes. Thus, the procedure assigns each neighbourhood an income that reflects an identical position to that it had in time *t*; also, the vector will maintain the same shape of distribution (i.e. the same level of inequality) as the vector of incomes at time *t*.
4. The ‘inequality’ counterfactual vector applies the Lorenz curve of the vector of incomes observed at time *t + 1* to the vector of incomes observed at time *t*. This is done by constructing a ‘structural’ counterfactual vector (as described in article 2 above), while eliminating the growth factor by also applying the inverse function to that described in (3). This way, the vector of neighbourhoods will reflect the same positional order as in time t, and the same overall level of incomes, and only manifest the effect of inequality that occurred in the transition between *t* and *t + 1.*

The next step in this approach is to quantify the total amount of change, and the amount associated with each counterfactual vector using a measure of mobility. Total change is computed using the initial and final observed vectors of neighbourhood average income. Component contributions are separately computed by using each counterfactual vector instead of the observed vector of final incomes. Van Kerm (2004) listed several applicable mobility measures, which comply with required axioms. Among them, we chose to use the measure proposed by Fields and Ok (1999b). Its advantage is that it can be simply disaggregated to reflect the contributions of groups of neighbourhoods. The measure is defined as:

$$ {m}_n\left(x,y\right)=\frac{1}{n}\sum \limits_{i=1}^n\left|\log {y}_i-\log {x}_i\right| $$

where (in the context of this application), *y*_*i*_ and *x*_*i*_ are neighbourhood average incomes at the final and initial observation periods respectively, and *n* refers to the number of neighbourhoods. This measure conforms to a concept of movement that focuses on the distance between final and initial values given in absolute terms (referring to dollar income). Due to the absolute value notation the measure would represent the average movement of incomes among neighbourhoods regardless of the directions of change; otherwise, the exchange component would sum up to zero. In computing contributions of neighbourhood sub-groups, a directional measure should be used by omitting the absolute value bars in the equation presented above. This variant of the mobility measure would reflect both the magnitude and direction of change experienced by each individual neighbourhood or group of neighbourhoods.

Finally, Van Kerm (2004) referred to a problem that different sequences of eliminating factors from the total mobility measure result in different component contributions. While each computation represents the marginal impact of each factor, they do not sum up to the total mobility computed. In order to derive additive components, Van Kerm proposed to use the Shapley decomposition procedure [see Shorrocks (2013, based on a previous version from 1999) for a comprehensive presentation]; this procedure averages the contributions computed by applying each possible sequence of elimination.

## Components of Neighbourhood Change in Metropolitan US – An Empirical Example

To demonstrate the decomposition of total neighbourhood change into its contributing components, we use the Longitudinal Tract Data Base (LTDB) which provides data on all US census tracts within constant 2010 boundaries for the years 1970–2010. The database was processed to allow for geographical consistency in longitudinal analysis, by Spatial Structures in the Social Sciences at Brown University (Logan et al. 2014). We focus on median household incomes for the years 1980 and 2010 which are based on sample counts (the long-form questionnaire of the 1980 census and the American Community Survey for 2010). The 1980 data is specified in real 2010 dollars. In order to offer a comparative view on the components of socioeconomic change in different places, we examined 22 of the largest metropolitan areas in the US as of 2010, after excluding those for which data of more than 10% of neighbourhoods were missing at either 1980 or 2010.

**Fig. 3:**
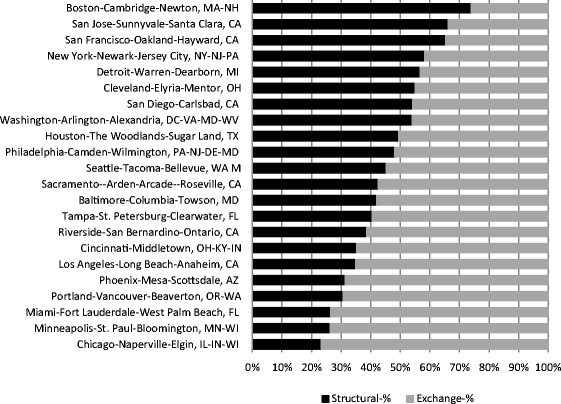
The relative shares of ‘exchange’ and ‘structural’ processes in total neighbourhood change in 22 US metropolitan areas

Figure 3 shows the relative contributions of ‘exchange’ and ‘structural’ factors to the average total change experienced by neighbourhoods of each metropolitan area. At a first glance, one can notice the large variation among metropolitan areas in the relative roles of change components. The ‘exchange’ component accounted for 74%–77% of total change in Chicago, Minneapolis and Miami. In Boston, San Jose and San Francisco, however, ‘Structural’ components were dominant, accounting for 74%, 66% and 65% of change respectively. A most substantial amount of change is driven, therefore, by higher-level processes that affect the distribution of neighbourhoods within each metropolitan area. These shares illustrate how by focusing exclusively on urban-level dynamics a notable part of change experienced by neighbourhoods can be overlooked.

**Fig. 4:**
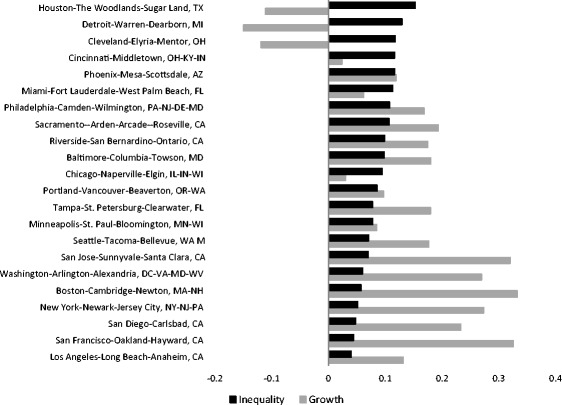
Absolute change associated with growth/decline and increasing inequality in 22 US metropolitan areas

The effect of the two structural processes, ‘growth/decline’ and ‘inequality’, on the average neighbourhood income also varies remarkably among different metropolitan areas (Fig. 4). The first thing to notice is the negative association between the effects of overall growth and inequality. Neighbourhoods in metropolitan areas which were most affected by overall growth were the least affected by increasing inequalities. These metropolitan areas encompass ‘superstar’ cities such as San-Francisco, New-York and Boston, that are characterized by exploding demand coupled with a constrained supply that make them the most expensive places (Gyourko et al. 2006). In these cities, increasing inequality has not been a substantial factor of neighbourhood change, because low- and middle income households have been gradually priced out of the increasingly unaffordable metropolitan housing markets (Florida 2017; Gyourko et al. 2006), and so the inequality among neighbourhoods within the metro area did not increase much. This process, however, has most likely involved increasing disparities between the metropolitan area and the surrounding region, such that the increasing inequality was ‘absorbed’ at a higher spatial level.

Neighbourhoods in metropolitan areas at the middle range of Fig. 4, such as Baltimore, Philadelphia and Sacramento, experienced a more moderate growth of incomes but more significant changes driven by the inequality factor. Following the same theoretical reasoning of Gyourko et al. (2006), in those cities the increasing demand was probably not high enough to spur gentrification processes all over the metropolitan area; low-income households most likely remained in previously low-income neighbourhoods and high-income neighbourhoods absorbed the demand such that disparities among low- and high- income neighbourhoods increased. Finally, there are metropolitan areas whose neighbourhoods experienced overall income decline (or very little growth), and were also very much affected by increasing inequality. These characteristics, however, are shared by two different types of places; those which were shrinking in population, such as Detroit, Cleveland and Cincinnati, and those which were expanding such as Houston. In the former, the overall decline in incomes reflects the direct effect of deindustrialization on employment and income, and the plummeting demand to living in these cities. The large role of inequality in neighbourhood change reflects the uneven spread of decline across metropolitan neighbourhoods, for example in Detroit, where the richest neighbourhoods were left almost intact (Guerrieri et al. 2012). Although the ‘growth’ and ‘inequality’ factors of neighbourhood change in Houston are quite similar in extent to those of Rust-Belt metropolitan areas, Houston’s underlying story is different. It is one of the fastest growing metropolitan areas in the US, with a regulatory environment that enables an abundant supply of affordable housing; this drew middle to low-income households, among which many immigrants (Center for opportunity urbanism 2016).

**Fig. 5:**
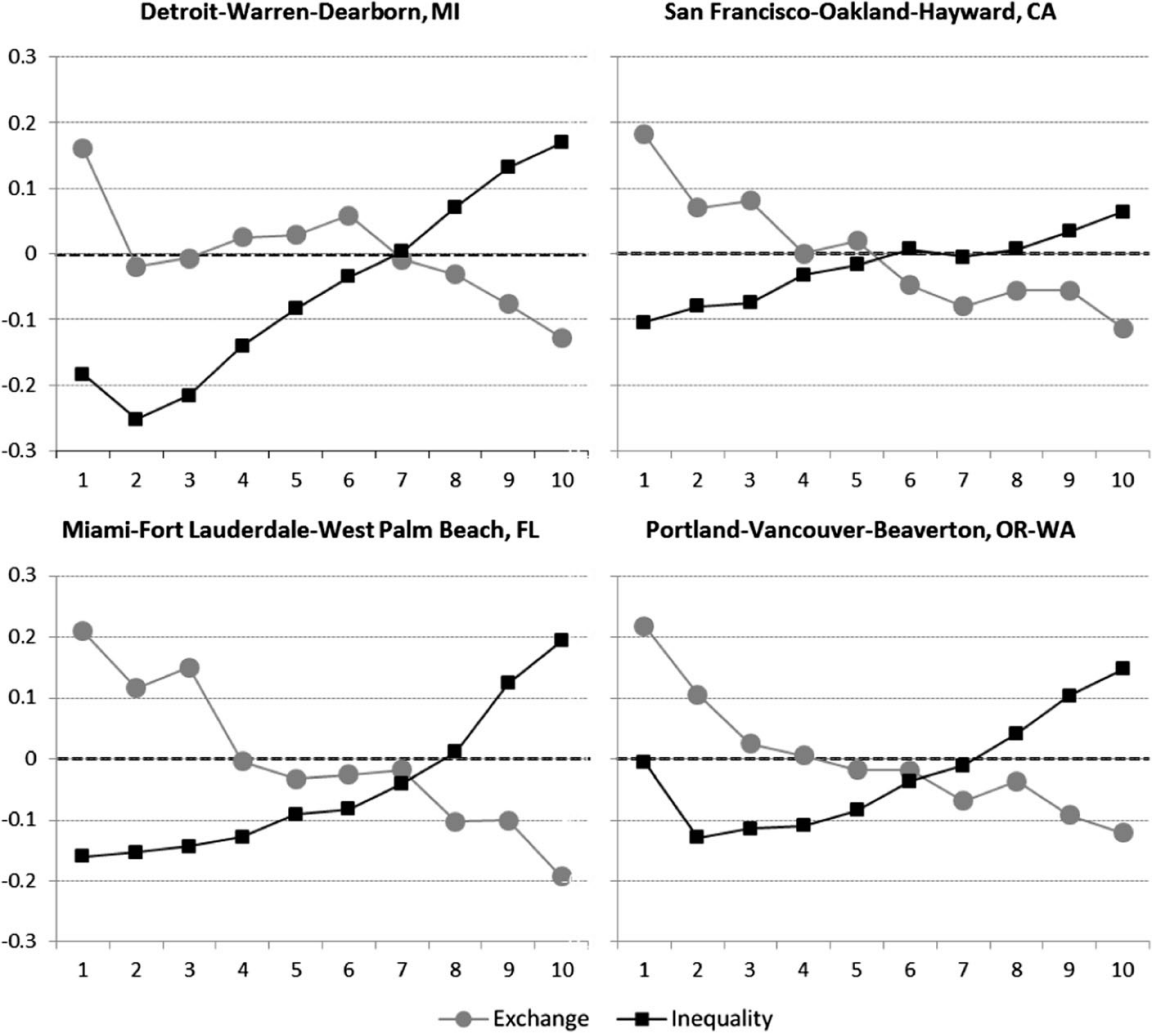
Absolute change experienced by neighbourhoods of different income deciles (1 = lowest decile) in selected metropolitan areas, due to exchange processes and increasing inequality

The role of inequality in neighbourhood socioeconomic change, which has been practically ignored to date, is actually substantial. If we disregard the few ‘superstar’ cities in which the inequality factor does not significantly generate neighbourhood change at the intra-metropolitan level, we see that the contribution of that factor is at least 10% of the total. It can amount to a fifth of total change in ‘middle-range’ metropolitan areas such as Miami and Chicago. It accounts for a third of total change in Cincinnati neighbourhoods and more than a half in Houston, Detroit and Cleveland. These figures demonstrate that the effect of inequality is by no means negligent. If this effect is confounded with the exchange effect by the use of relative measures, then empirical analyses risk drawing the wrong conclusions about what drives neighbourhood socioeconomic change. This becomes more evident when examining how the exchange and inequality factors affect neighbourhoods that differ by their socioeconomic starting position, that is their income decile in 1980 (Fig. 5). Conforming to the expectations, exchange processes follow a mean reversion pattern; high-income neighbourhoods generally decrease and low-income ones increase. This pattern is evident in all metropolitan areas, four of which are presented in the figure. Increasing inequality affects neighbourhoods in an opposite manner; it makes high income neighbourhoods increase and low-income ones decrease. When the increase in inequality is substantial, it more than offsets the changes neighbourhoods experienced due to the exchanges of relative positions, as the case of Detroit demonstrates. In places like San-Francisco the effect of inequality is lower such that it only attenuates the exchange effect.

**Fig. 6:**
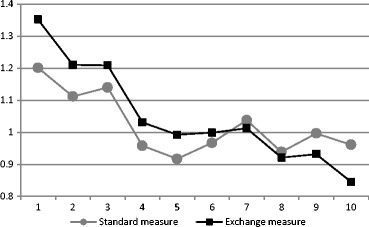
Absolute change experienced by neighbourhoods of different income deciles (1 = lowest decile) in Miami, FL metropolitan area: A comparison of a ‘standard’ measure of neighbourhood change which incorporates the effect of increasing inequality and an ‘exchange’ measure which leaves out the ‘inequality’ effect

A final analysis shows exactly how the standard relative measure of neighbourhood change underestimates the amount of change that can be attributed to urban-level processes. Drawing on the Miami Metropolitan area as an illustration, we computed the amount of change experienced by neighbourhoods of differing initial positions in 1980, using the common relative measure and a corresponding measure that represents the ‘exchange’ factor exclusively (Fig. 6). The first measure, as for example used by Rosenthal (2008), is based on computing the 2010 ratio of neighbourhood average income to the average for all neighbourhoods in the respective metropolitan area, and dividing it by the respective 1980 ratio. This measure excludes the ‘growth’ component but captures both the ‘exchange’ and ‘inequality’ components. The second measure is computed by dividing each neighbourhood’s ‘exchange-driven’ income from the ‘exchange’ counterfactual vector by the initial observed income in 1980. So, it is essentially the same ratio measure as the first, but it excludes the effect of increasing inequality among neighbourhoods. The plot of the two measures illustrates how the inclusion of the inequality factor in the common relative measure of change moderates the change gradient; low-income neighbourhoods seem to have improved their positions much less than in reality, and high-income neighbourhoods seem to have worsened much less. With no increase in the inequality among neighbourhoods the two measures would coincide.

## Discussion

This paper presents a new application to the measurement and analysis of neighbourhood socioeconomic change. The application makes a distinction between the contributions of different processes that drive these changes; we classify them as ‘exchange’ processes which refer to urban-level dynamics that generate change in neighbourhoods’ urban-relative statuses, and as ‘structural’ processes that operate at higher levels and change neighbourhoods by affecting the overall socioeconomic composition of urban areas. The two different structural processes are overall income growth or decline (‘growth/decline’ effect) that can translate into the growth or decline of the average income of neighbourhoods (Andersson and Hedman 2016; Galster et al. 2003; Zwiers, Bolt, et al. 2016), and changes in the inequality among individuals (‘inequality’ effect) that can translate into changing inequality among neighbourhoods. Most studies on neighbourhood change have measured neighbourhood change based on their status relative to other neighbourhoods in the respective urban area (Choldin et al. 1980; Choldin and Hanson 1982; Delmelle 2015; Fogarty 1977; Gould Ellen and O’Regan 2008; Landis 2016; Logan and Schneider 1981; Owens 2012; Rosenthal 2008; Rosenthal and Ross 2015). Relative measures neutralize the growth/decline effect, such that the actual extent of change in neighbourhoods’ conditions is overlooked. Also, these measures confound the effect of urban-level ‘exchange’ processes with structural ‘inequality’ processes. This paper therefore suggests an alternative approach in the measurement of neighbourhood change. An approach that on the one hand, will enable to take account for the overall amount of change in neighbourhood socioeconomic conditions, and on the other hand, will enable to make a distinction between the different contributing processes.

The proposed approach is based on the distinction among similar process components in social and income mobility research. The paper applies a method presented by Van Kerm (2004) in the context of income mobility, which can be applied to various mobility measures. By applying Van Kerm’s decomposition to two different variants of a measure of mobility suggested by Fields and Ok (1999b), the presented approach involves two levels of analysis which may provide complementary insights in the context of neighbourhood change. The first level of analysis is the ‘system’ level which refers to the urban or metropolitan area as a whole. At this level, the total amount of change occurring among metropolitan neighbourhoods is decomposed to reflect the amount of change that can be attributed to ‘exchange’ and the two ‘structural’ effects (growth/decline and inequality). The second level of analysis can provide insight into the effect of different factor components on different parts of the neighbourhood income distribution, different neighbourhood types or neighbourhoods located at different places.

We demonstrated the approach in an empirical analysis of components of neighbourhood socioeconomic change across 22 metropolitan areas in the US during the period 1980–2010, using the Longitudinal Tract Data Base (Logan et al. 2014). The analysis indicates that the relative roles of structural and exchange processes vary remarkably. In almost half the cases, structural processes accounted for half or more of the total income change experienced by neighbourhoods. Also the ‘Growth/decline’ and ‘inequality’ factors interact very differently across different metropolitan areas. In general, factor contributions indicate that the larger the effect of growth, the lesser the effect of inequality among metropolitan neighbourhoods. This finding can be explained by theories that describe how demand coupled with constrained housing supply increase incomes, decrease affordability and therefore decrease neighbourhood inequality within the metropolitan area (Gyourko et al. 2006). In addition, the analysis emphasizes how increasing inequality and urban-level dynamics offset each other. This implies that low-income neighbourhoods that improve their positions in the urban hierarchy may not experience any actual income change because of the inequality effect. From a policy perspective, this exemplifies the potential role of people-based policies (as opposed to place-based policies) in tackling urban deprivation. Finally, the analysis demonstrates how common relative measures which include the effect of inequality underestimate, as consequence, the amount of socioeconomic change that should actually be attributed to urban-level processes.

By using the proposed method to account for exchange and structural processes in neighbourhood socioeconomic change, empirical studies can disentangle the complexity often observed among single urban areas and across them. As most cities struggle with changing disparities among neighbourhoods, and with evolving spatial patterns of these disparities, questions often arise regarding the underlying dynamics; for example, is gentrification of inner city areas merely a changing centre of gravity, or is it a result of the whole city becoming wealthier? Is increasing poverty incidence among peripheral neighbourhoods related to the relocation of the poor due to a cycle of urban development or is it related to changing overall inequality?

Such questions gain importance given the global trends in inequality. Neighbourhoods, to a large extent now, reflect the story of increasing overall inequality which translates into increasing disparities within and among cities. In this context, it seems no longer plausible to ignore the role of inequality in neighbourhood change processes. What happens to neighbourhoods within the isolated context of each city or metropolitan area is a very incomplete picture of change processes. It may be difficult to accept a conclusion that many neighbourhoods are stable over the long run (as was concluded for example by Delmelle 2017) with only their urban-relative positions are taken into account, while neighbourhoods increase or decrease substantially because of structural processes. The fact that neighbourhoods preserve their relative standings does not necessarily render them stable. A neighbourhood in San-Francisco, Boston, New-York and other cities can keep a similar position within the city’s hierarchy, but at the same time be twice as rich compared to a few decades ago. Similarly, low-positioned neighbourhoods in Cleveland experienced much extremer deprivation following structural transformations, even if they maintained their relative status. The method and illustration presented in this paper exemplify that neighbourhoods cannot be viewed as only part of their respective urban systems; they are deeply embedded in higher-level contexts and are greatly affected by the contemporary reality of increasing inequalities at multiple spatial scales. This understanding should be reflected in how neighbourhood change is measured and analysed.

## Footnotes

1 As McClendon (1977) himself noted in footnote 3, p. 60.
